# Activated protein C, severe sepsis and 28-day mortality

**DOI:** 10.1186/cc10630

**Published:** 2012-03-20

**Authors:** M De La Torre-Prados, A García-de la Torre, M Nieto-González, I Lucena-González, R Escobar-Conesa, A García-Alcántara, A Enguix-Armada

**Affiliations:** 1 Hospital Virgen de la Victoria, Málaga, Spain

## Introduction

Protein C (PC) deficiency is prevalent in severe sepsis, studies showing that more than 80% of patients with severe sepsis have a baseline PC level below the lower limit of normal [1,2]. The aim of the study was to relate the anticoagulation activity evaluated by PC, with clinical parameters and 28-day mortality.

## Methods

A cohort study of 150 patients >18 years with severe sepsis according to the Surviving Sepsis Campaign, in an ICU of a university hospital. Demographic, clinical parameters and coagulation markers during the first 24 hours were studied. PC activity was analysed using a haemostasis laboratory analyser (BCS^® ^XP; Siemens). Descriptive and comparative statistical analysis was performed using SPSS version 15.0 (SPSS Inc., Chicago, IL, USA).

## Results

We analyzed 150 consecutive episodes of severe sepsis (16%) or septic shock (84%) admitted to the ICU. The median age was 64 years old (interquartile range, 48.7 to 71); male: 60%. The beginning of severe sepsis took place in the emergency area in 46% of cases. The main sources of infection were respiratory tract 38% and intra-abdomen 45%; 70.7% had medical pathology. The 28-day mortality was 22.7%. The profile of death patients were men (64.7%, *n *= 22), with significantly higher average age (63 vs. 57 years; *P *= 0.049), as well as clinical severity scores, APACHE II (29.8 vs. 24.1; *P *< 0.001) and SOFA (12.1 vs. 8.9; *P *< 0.001) and major dysfunction organs (4.6 vs. 3.6; *P *< 0,001); we observed significantly major consumption of PC (55.2 vs. 70.1, *P *= 0.011). Lower levels of PC were found in surgery septic shock patients, neurological focus or catheter-related infection and Gram-negative pathogens from blood cultures. The ROC analysis showed superior risk prediction of SOFA score for 28-day mortality, AUC 0.81 (95% CI: 0.73 to 0.88, sensitivity: 73.5%; specificity: 76.7%, *P *= 0.001), that improves by combining with PC, AUC 0.83 (95% CI: 0.75 to 0.90, sensitivity: 77%; specificity: 83%, *P *= 0.001).

## Conclusion

This cohort study showed an improvement in the survival in septic patients under a lower consumption of PC. Low levels of PC are associated with more severity in Sepsis, dysfunction organ and poor outcome.
